# Progression and Prognosis of Paravalvular Regurgitation After Transcatheter Aortic Valve Implantation

**DOI:** 10.5935/abc.20170172

**Published:** 2017-12

**Authors:** Rafael Alexandre Meneguz-Moreno, Antônio de Castro-Filho, Auristela Isabel de Oliveira Ramos, Mayra Zumarraga, David Le Bihan, Rodrigo Barretto, Dimytri Alexandre de Alvim Siqueira, Alexandre Antonio Cunha Abizaid, Amanda Guerra de Moraes Rego Sousa, J. Eduardo Sousa

**Affiliations:** 1 Instituto Dante Pazzanese de Cardiologia, São Paulo, SP; - Brazil; 2 Universidade Federal de Sergipe, Lagarto, SE; - Brazil; 3 Hospital do Coração da Associação Sanatório Sírio, São Paulo, SP- Brazil

**Keywords:** Aortic Valve Insufficiency / complications, Heart Valve Prosthesis Implantation, Prognosis, Mortality

## Abstract

**Background:**

The impact of paravalvular regurgitation (PVR) following transcatheter aortic valve implantation (TAVI) remains uncertain.

**Objective:**

To evaluate the impact of PVR on mortality and hospital readmission one year after TAVI.

**Methods:**

Between January 2009 and June 2015, a total of 251 patients underwent TAVI with three different prostheses at two cardiology centers. Patients were assessed according to PVR severity after the procedure.

**Results:**

PVR was classified as absent/trace or mild in 92.0% (n = 242) and moderate/severe in 7.1% (n = 18). The moderate/severe PVR group showed higher levels of aortic calcification (22% vs. 6%, p = 0.03), higher serum creatinine (1.5 ± 0.7 vs. 1.2 ± 0.4 mg/dL, p = 0.014), lower aortic valve area (0.6 ± 0.1 vs. 0.7 ± 0.2 cm^2^, p = 0.05), and lower left ventricular ejection fraction (49.2 ± 14.8% vs. 58.8 ± 12.1%, p = 0.009). Patients with moderate/severe PVR had more need for post-dilatation (p = 0.025) and use of larger-diameter balloons (p = 0.043). At one year, all-cause mortality was similar in both groups (16.7% vs. 12%, p = 0.08), as well as rehospitalization (11.1% vs. 7.3%, p = 0.915). PVR grade significantly reduced throughout the first year after the procedure (p < 0.01). The presence of moderate/severe PVR was not associated with higher one-year mortality rates (HR: 0.76, 95% CI: 0.27-2.13, p = 0.864), rehospitalization (HR: 1.08, 95% CI: 0.25-4.69, p=0.915), or composite outcome (HR: 0.77, 95% CI: 0.28-2.13, p = 0.613).

**Conclusion:**

In this sample, moderate/severe PVR was not a predictor of long-term mortality or rehospitalization. (Arq Bras Cardiol. 2017; [online].ahead print, PP.0-0)

## Introduction

Patients with symptomatic, severe aortic stenosis, at high risk for surgery, treated with transcatheter aortic valve implantation (TAVI), have shown a favorable outcome, as described in several randomized studies.^1-3^

Although the TAVI technique has reached relative maturity, paravalvular regurgitation (PVR) remains a possible complication. It is not well established whether or not the presence of PVR is directly associated with worse prognosis after TAVI or whether the relationship between PVR and TAVI is merely an association.^4^

In the PARTNER (Placement of aortic transcatheter valve) trial, the presence of moderate/severe PVR in inoperable patients had a negative impact on one-year all-cause mortality.^2^ On the other hand, in the group of patients at high risk, even the presence of mild PVR post-TAVI was associated with increased mortality.^5,6^ However, in another randomized study, the CoreValve U.S. Pivotal Trial,^3^ PVR severity decreased after one year, and only severe PVR was associated with mortality.

The present study aimed to evaluate the presence and the progression of PVR one year after TAVI, and its impact on adverse clinical outcomes.

## Methods

### Classification of aortic stenosis by echocardiography

Analysis of aortic valve area (AVA) and aortic regurgitation was performed by echocardiography in all patients using the multiparametric method according to published guidelines.^7,8^

### Patients’ selection and indication for procedure

For risk estimation, we used the STS^9^ (Society of Thoracic Surgeons) recommendations, the logistic EuroSCORE (European System for Cardiac Operative Risk Evaluation)^10^ and the EuroSCORE II.^11^

All symptomatic patients with severe aortic stenosis (valve area ≤ 1.0 cm^2^), at high surgical risk, who had undergone TAVI in two excellence centers in cardiology in Brazil between January 2009 and June 2015 were included in this analysis. The multidisciplinary team was similar in both centers.

All data were collected from the institutions’ databases using standardized forms developed for the study, and organized in spreadsheets.

Assessment of clinical and echocardiographic data was performed at 30 days, 6 months and 1 year, during medical visits and telephone contact, according to the clinical routine of each center.

Patients were included in this prospective study after signing the informed consent form. The protocol was approved by the Research Ethics Committee of each institution according to the Helsinki declaration.

### Implantation technique and procedures

The self-expanding, percutaneous CoreValve (Medtronic, Minneapolis, USA) prosthesis, the Acurate (Symetis SA, Lausanne, Switzerland) prosthesis, or the balloon-expandable Edwards Sapien-XT (Edwards Lifesciences, Irvine, EUA) valve prosthesis were used, at the interventional cardiologist’s discretion.

Most procedures were performed under general anesthesia and with transesophageal echocardiography. Transfemoral vascular access was indicated in all patients who had a favorable vascular access. Arterial hemostasis was performed using a specific device, mediated by the Perclose ProGlide® Suture-Mediated Closure System (Abbott Vascular™, Santa Clara, USA) or surgical access. When transfemoral access was not possible, the transapical, transaortic or the subclavian accesses were used as alternatives. Both predilatation and postdilatation were performed at the intervention team’s discretion. Whenever possible, patients were extubated in the operating room and kept in observation in the intensive care unit during 24-48 hours. Hospital discharge occurred according to patient’s clinical progress after TAVI. Hemodynamic data were obtained during the TAVI procedure and by echocardiography before hospital discharge.

### Definitions

The use of TAVI device was considered successful if the prosthesis was correctly implanted, without a prosthesis-patient mismatch, with an aortic valve mean gradient < 20 mmHg and absence of moderate or severe aortic regurgitation, according to echocardiography results.

Primary outcomes were defined according to the Valve Academic Research Consortium (VARC-2) criteria^12,13^ and systematically evaluated by two experienced cardiologists. Primary outcome was established by an outcome composed of global mortality and rehospitalization due to cardiac causes. Secondary outcomes were death for cardiac reasons, NYHA (New York Heart Association) classification for dyspnea, acute myocardial infarction and stroke.

### Clinical follow-up

Clinical and echocardiographic follow-up was performed at 30 days after discharge and every six months.

Dual antiplatelet therapy was started with a loading dose of acetylsalicylic acid (ASA) and clopidogrel 24 hours before TAVI procedure; clopidogrel at 75 mg/day was maintained up to 6 months thereafter and ASA 100mg/day was continuously maintained.

### Echocardiographic follow-up

Evaluation of the aortic prosthesis was performed according to the Valve Academic Research Consortium (VARC-1), the American Society of Echocardiography, and the European Society of Echocardiography criteria.^1,8,14,15^

Echocardiography was performed by two experienced technicians, and patients were classified according to PVR degree as ‘absent/trace’, ‘mild’, ‘moderate’ or ‘severe’ regurgitation, using a semiquantitative criteria, as previously described by Hahn et al.^16^

### Statistical analysis

Continuous variables were described as mean and standard deviation, and compared using the one-way ANOVA after being tested for normality by the Shapiro-Wilk test. Categorical variables were described as absolute numbers and percentage, and were analyzed by the chi-squared test or the Fisher exact test, as appropriate. For analysis of PVR progression based on post-TARVI PVR, distribution homogeneity in each PVR subgroup over time was tested using the Stuart-Maxwell test (generalized McNemar test). Survival analysis was performed using the Kaplan-Meier method, and the difference between the PVR subgroups was compared using the log-rank test. A p < 0.05 was considered statistically significant. Analyses were performed using the R program version 3.1 (The R Foundation for Statistical Computing, Vienna, Austria) and the SPSS (Statistical Package for the Social Science, Chicago, EUA) program version 20.

## Results

### Patients

A total of 259 patients underwent TAVI during the study period. Six patients died during the procedure and two patients were lost to follow-up, and hence, excluded from the study. Among the remaining 251 patients, the echocardiographic study performed before hospital discharge identified 18 patients (7.1%) with moderate PVR (group 1) and 233 patients (92.8%) in Group 2, with absent/trace of PVR (n = 145) or mild PVR (n = 88). There was no case of severe PVR in the sample.

Mean age of participants was 82.16±6.70 years, and more than half of patients (55.5%) were women. In 224 patients (89.2%), TAVI was performed via the transfemoral access. Mean STS score was 6.62 ± 4.78%, and 78.9% had NYHA class III or IV heart failure. As compared with group 2, group 1 showed a higher degree of aortic valve calcification than group 2 (22.0% vs. 6.0%; p=0.03), higher creatinine levels (1.53 ± 0.71 vs. 1.18 ± 0.43 mg/dL; p = 0.014), lower AVA (0.61±0.12 vs. 0.69 ± 0.17 cm^2^; p = 0.05) and more severe left ventricular dysfunction (49.17 ± 14.79% vs. 58.82 ± 12.14%; p = 0.009). Baseline characteristics are described in Table 1.

**Table 1:** Baseline characteristics

	All patients (n = 251)	Moderate/severe PVR (n = 18)	Absent / trace or mild PVR (n = 233)	p-value
Clinical characteristics				
Age (years)	82.16 ± 6.70	80.50 ± 7.96	82.28 ± 6.59	0.680
Female sex	138 (55.5%)	6 (33.3%)	132 (56.7%)	0.083
Weight (kg)	68.42 ± 12.87	67.33 ± 12.69	68.51 ± 12.91	0.592
BMI (kg/m^2^)	26.44 ± 4.49	25.43 ± 4.32	26.52 ± 4.50	0.320
Syncope/Presyncope	52 (20.7%)	5 (27.8%)	47 (20.2%)	0.442
DM	85 (33.9%)	5 (27.8%)	80 (34.3%)	0.571
Insulin-dependent DM	21 (8.4%)	1 (5.6%)	10 (8.6%)	0.654
COPD	36 (14.3%)	2 (11.1%)	34 (14.6%)	0.684
Dyslipidemia	159 (63.3%)	12 (66.7%)	147 (63.1%)	0.761
Hypertension	208 (82.9%)	15 (83.3%)	193 (82.8%)	0.956
Smoking	44 (17.5%)	4 (22.2%)	40 (17.2%)	0.586
PVD	54 (21.5%)	4 (22.2%)	50 (21.5%)	0.939
Carotid artery disease	44 (17.5%)	4 (22.2%)	40 (17.2%)	0.586
Atrial fibrillation	2 (0.8%)	1 (5.6%)	1 (0.4%)	0.138
Previous stroke	14 (5.6%)	1 (5.6%)	13 (5.6%)	0.996
Neurologic sequelae	13 (5.2%)	1 (5.6%)	12 (5.2%	0.940
CAD ≥ 50%	131 (52.2%)	9 (50%)	122 (52.4%)	0.846
CABG	52 (20.7%)	4 (22.2%)	48 (20.6%)	0.771
Previous PCI	62 (24.7%)	3 (16.7%)	59 (25.3%)	0.573
AMI	43 (17.1%)	5 (27.8%)	38 (16.3%)	0.213
AMI < 30 days	3 (1.2%)	1 (5.6%)	2 (0.9%)	0.07
Previous pacemaker	28 (11.2%)	1 (5.6%)	27 (11.6%)	0.702
Previous ICD	2 (0.8%)	0	2 (0.9%)	0.861
NYHA				0.841
I	11 (4.5%)	0	11 (4.9%)	
II	41 (16.8%)	2 (11.1%)	39 (17.3%)	
III	159 (65.2%)	14 (77.8%)	145 (64.2%)	
IV	33 (13.5%)	2 (11.1%)	31 (13.7%)	
Porcelain aorta	13 (5.2%)	2 (11.1%)	11 (4.7%)	0.238
Previous valve repair surgery	17 (6.8%)	3 (16.7%)	14 (6.0%)	0.111
Creatinine (mg/dL)	1.21 ± 0.47	1.53 ± 0.71	1.18 ± 0.43	0.014
GFR (mL/min.1.73m^2^)	42.97 ± 26.58	38.3 ± 13.53	43.3 ± 27.39	0.374
Creatinine cl < 50 mLmin.1.73m^2^	142 (56.6%)	14 (77.8%)	128 (54.9%)	0.05
Log. EuroSCORE (%)	21.5 ± 11.96	25.17 ± 13.26	21.21 ± 11.83	0.211
EuroSCORE II (%)	7.57 ± 6.30	7.5 ± 6.19	8.34 ± 7.77	0.849
STS (%)	6.62 ± 4.78	6.35 ± 2.46	6.64 ± 4.92	0.462
**Echocaridographic variables**				
AVA (cm^2^)	0.69 ± 0.17	0.61 ± 0.12	0.69 ± 0.17	0.056
Aortic valve annulus (cm^2^)	22.32 ± 5.25	20.93 ± 8.99	22.43 ± 4.86	0.287
LVEF (%)	58.11 ± 12.58	49.17 ± 14.79	58.82 ±12.14	0.009
Mean gradient (mmHg)	53.58 ± 15.62	52.44 ± 20.51	53.67 ± 15.22	0.471
Maximum gradient (mmHg)	86.45 ± 23.34	85.94 ± 31.24	86.49 ± 22.7	0.701
Continuation				
LVEDD (mm), mean ± DP	50.81 ± 7.28	54.76 ± 8.04	50.51 ± 7.16	0.030
PAP (mmHg), mean ± DP	49.14 ± 14.0	51.18 ± 13.22	48.97 ± 14.08	0.451
Extensive calcification of the aorta valve	18 (7.2%)	4 (22.2%)	14 (6.0%)	0.030
Degree of aortic regurgitation ≥ 2	25 (7.6%)	3 (16.7%)	16 (6.9%)	0.297
Degree of mitral regurgitation ≥ 2	47 (17.5%)	5 (27.8%)	42 (16.7%)	0.395

Data expressed as mean ± SD or absolute number and percentage as appropriate. AMI: acute myocardial infarction; AVA: aortic valve area; BMI: body mass index; CAD: coronary artery disease; Creat cl: creatinine clearence calculated by the Cockroft-Gault formula; COPD: chronic obstructive pulmonary disease; DM: diabetes mellitus; EuroSCORE: European system for cardiac operative risk evaluation; ICD: implantable cardioverter defibrillator; LVEDD: left ventricular end diastolic diameter; LVEF: left ventricular ejection fraction; CABG: coronary artery bypass graft; PAP: pulmonary artery pressure; PCI: percutaneous coronary intervention; PVD: peripheral vascular disease; GFR: glomerular filtration rate; STS: Society of Thoracic Surgeons; NYHA: New York Heart Association functional class.

Patients with moderate/severe PVR had a greater need for postdilatation (p = 0.025), and for using larger-diameter balloons (p = 0.043). Characteristics of the TAVI procedure are described in Table 2.

**Table 2:** Data of transcatheter aortic valve implantation procedure

	All patients (n = 251)	Moderate/severe PVR (n = 18)	Absent/trace or mild PVR (n = 233)	p-value
Time of procedure (min)	97.55 ± 47.32	125.06 ± 39.97	95.41 ± 47.25	0.002
Contrast volume (mL)	128.31 ± 74.32	123.75 ± 35.19	128.64 ± 76.45	0.479
Type of prosthesis				0.350
Sapien-XT	96 (38.3%)	4 (22.2%)	92 (39.5%)	
CoreValve	95 (37.8%)	9 (50%)	86 (36.9%)	
Acurate	60 (23.9%)	5 (27.8%)	55 (23.6%)	
General anesthesia	242 (96.4%)	18 (100%)	224 (96.1%)	0.395
Access route				0.680
Femoral	224 (89.2%)	17 (94.4%)	207 (88.8%)	
Transapical	10 (4.0%)	0	10 (4.3%)	
Transaortic	13 (5.2%)	1 (5.6%)	12 (5.2%)	
Subclavian	1 (0.4%)	0	1 (0.4%)	
Hemostatic compression device	170 (75.7%)	13 (72.2%)	177 (76%)	0.792
Valve repair before or during the procedure	185 (73.7%)	16 (88.9%)	169 (72.5%)	0.128
Valve repair before or during the procedure (balloon diameter)	19.31 ± 6.37	18.17 ± 8.48	19.42 ± 6.14	0.556
Post-dilatation	80 (31.9%)	10 (55.6%)	70 (30%)	0.025
Post-dilatation (balloon diameter)	23.19 ± 1.94	24.2 ± 0.92	23.04 ± 2	0.043
New permanent pacemaker	29 (11.6%)	2 (11.1%)	27 (11.6%)	0.953
**Post-procedure echocardiographic variables**				
AVA (cm^2^)	1.88 ± 0.29	1.89 ± 0.23	1.88 ± 0.30	0.742
LVEF (%)	58.61 ± 13.54	51.56 ± 19.73	59.2 ± 12.77	0.175
LVEDD (mm)	49.48 ± 10.53	51.94 ± 15.11	49.26 ± 10.07	0.027
Mean gradient (mmHg)	10.70 ± 4.70	11.00 ± 3.79	10.67 ± 4.77	0.684
Maximum gradient (mmHg)	20.18 ± 8.30	20.56 ± 7.28	20.15 ± 8.40	0.847
PAP (mmHg)	45.83 ± 16.74	53.67 ± 18.78	45.05 ± 16.37	0.225

Data expressed as mean ± SD or absolute number and percentage, as appropriate. AVA: aortic valve area; LVEDD: left ventricular end diastolic diameter; LVEF: left ventricular ejection fraction; AMI: acute myocardial infarction; PAP: pulmonary arterial pressure.

### Follow-up

**Figure 1:**
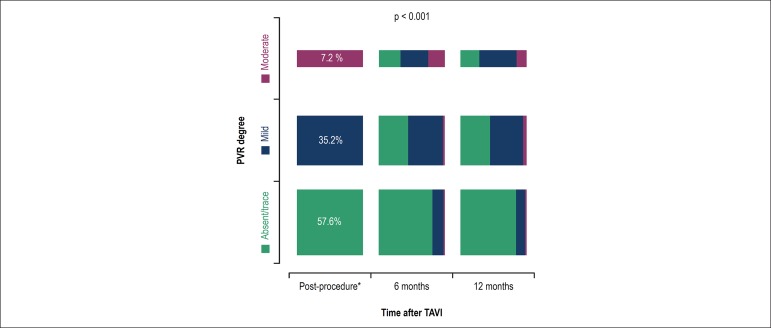
Distribution of patients with different paravalvular regurgitation (PVR) severity grades according to serial echocardiography analysis immediately after the procedure and at 6 months and 12 months after transcatheter aortic valve implantation (TAVI) *echocardiography before hospital discharge

At the end of one year, 134 patients had two echocardiographic analyses (post-TARVI and at one year); 111 patients (82.8%) showed an improvement of PVR grade or no changes (p < 0.01), and 23 (17.1%) patients had a worsening of PVR (Figure 1). Of 18 patients with moderate and severe PVR before hospital discharge, 16 (88.9%) showed an improvement of at least one grade at one year of follow-up, and no patient had severe PRV.

**Figure 2:**
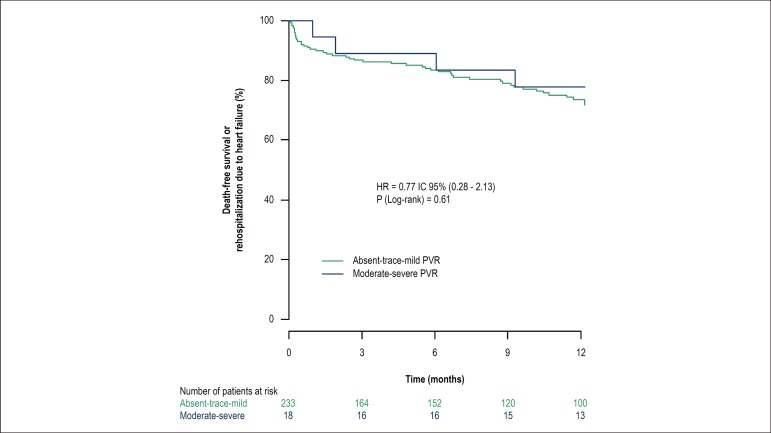
Kaplan-Meier curves showing the comparison of cumulative death-free survival or necessity of rehospitalization due to cardiac causes over the first year after transcatheter aortic valve implantation (TAVI) in patients with absent/trace or mild paravalvular regurgitation (PVR) in comparison with patients with moderate to severe PVR

Mean follow-up period was 13.2 months (interquartile range: 1.15-13.08). At the end of 1 year, all-cause mortality (16.7% vs. 12.0%; p = 0.081) and rehospitalization due to cardiac causes (11.1% vs. 7.3%; p = 0.915) were similar in both groups. There was no significant difference in all-cause mortality (RR: 0.76; 95%CI: 0.27-2.13; p = 0.864), rehospitalization due to cardiac causes (RR: 1.08; 95%CI: 0.25-4.69; p = 0.915) or composite outcome (RR: 1.06; 95%CI: 0.54-2.06; p = 0.873) (Figure 2). Also, no differences were found in the other clinical outcomes between the groups after 1 year (Table 3).

**Table 3:** Event rate one year after transcatheter aortic valve implantation

	All patients (n = 251)	Moderate/severe PVR (n = 18)	Absent/trace or mild PVR (n = 233)	p-value
Events				
Composite primary outcome	54 (21.5%)	4 (22.2%)	50 (21.5%)	0.614
All-cause mortality	31 (12.4%)	3 (16.7%)	28 (12.0%)	0.811
Death from cardiovascular causes	22 (8.8%)	3 (13.6%)	19 (8.2%)	0.218
Rehospitalization due to cardiovascular causes	19 (7.6%)	2 (11.1%)	17 (7.3%)	0.915
Stroke	8 (3.2%)	1 (5.6%)	7 (3.0%)	0.124
AMI	2 (0.8%)	0	2 (0.9%)	1.000

Data expressed as mean ± SD or absolute number and percentage, as appropriate. AMI: acute myocardial infarction; TAVI: transcatheter aortic valve implantation.

**Figure 3:**
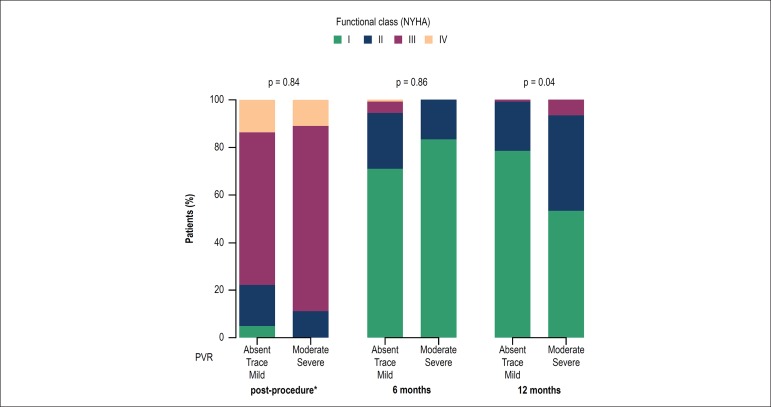
Bar graph showing the distribution of patients with different paravalvular regurgitation (PVR) grades and NYHA (New York Heart Association) functional class immediately after the procedure and at 6 months and 12 months after transcatheter aortic valve implantation (TAVI) *echocardiography before hospital discharge

With respect to dyspnea symptoms, no differences were detected between the NYHA groups at six months (p = 0.861), whereas at the end of one year, the group of patients with moderate/severe PVR were more symptomatic (0.047) (Figure 3). Considering only the functional classes III and IV, no differences were found between groups 1 and 2 at 6 months (0% in group 1 *vs*. 4.7% in group 2, p = 0.99) or at 1 year (6.7% in group 1 *vs*. 0.9% in group 2; p = 0.22) between groups 1 and 2.

## Discussion

Analysis of this sample of patients added to the knowledge about PVR following TAVI: 1) despite relatively frequent, PVR occurs in mild degree in most of the cases (92.6%); 2) the echocardiographic findings showed a regression in PVR severity grade at the end of the first year; 3) our findings did not show a relationship between moderate/severe PVR and a worse prognosis, although these patients had more symptoms of heart failure at the end of one year.

The frequency of PVR after TAVI varies between studies (50-85%),^17^ particularly due to technical difficulties in the diagnosis and the learning curve, in addition to different modalities of imaging exams, including transthoracic and transesophageal echocardiography, angiography, computed tomography angiography and magnetic resonance.^18^ The largest meta-analysis on the theme reported an incidence of 7.4% of moderate to severe PVR, with the use of first-generation devices (Sapien-XT e CoreValve).^19^

With the use of more recent prosthesis, such as SAPIEN-3 (Edwards Lifesciences, Irvine, EUA) and CoreValve Evolut-R (Medtronic, Minneapolis, EUA), the incidence of moderate/severe PVR at 30 days was 2.0-3.4%.^20-22^ Such incidence tends to decrease, as the use of TAVI has been extended to lower-risk patients and included new, repositionable prostheses: the Edwards CENTERA (Edwards Lifesciences, Irvine, USA), JenaValve (JenaValve Technology Inc., Irvine, USA), Lotus Valve™ System (Boston Scientific, Massachusetts, USA) and Portico™ (St. Jude Medical Inc., Minnesota, EUA) prostheses, which involves new mechanisms aimed at reducing the incidence of PVR, such as anchorage mechanism, or sealing skirts in its lower part to conform to the irregular surfaces of the aortic annulus.

In patients with moderate/severe regurgitation, there was a greater need for post-dilatation and larger-diameter balloons, probably due to more severe valvular calcification and larger aortic annulus, in addition to longer procedure time, although we did not perform an analysis of independent predictors of moderate to severe PVR. In previous studies, larger aortic annulus^23^ and important calcification were associated with higher PVR rates after the procedure,^24^ which is corroborated by our results showing that the group 2 was composed of more severely impaired patients, with lower AVA, greater left ventricular dysfunction and worse renal function. Taken together, these findings suggest that PVR patients are more likely to be more severe patients prior to the TAVI procedure.

Moderate to severe PVR has been known to be associated with poor short- and long-term clinical outcomes.^6,17,25,26^ However, there are few data on PVR progression over time and its association with clinical outcomes and symptoms worsening. Studies with a longer follow-up have shown a reduction in moderate/severe PVR, but this effect may be attributed to death of more severe patients at higher risk. The reduction in the severity of regurgitation may also be related to aortic annulus remodeling, expansion of nitinol and change in the left ventricular geometry.^4,6,26,27^

In the CoreValve US Pivotal Trial,^3^ PVR improved over one year, and only severe PVR after TAVI was associated with increased mortality rates, which may be associated with aortic root remodeling. In that study,^3^ at one year after discharge, 44% of patients showed an improvement of PVR of at least one grade, and 18% of patients, most of them with mild PVR, showed a worsening of the condition. Similar to our results, in the study by Oh et al.,^27^ 83% of patients with moderate PVR patients improved in up to one grade after one year of follow-up. In the PARTNER study, 31.9% of patients had an improvement in PVR severity grade after two years.^16,28^

The association between symptoms according to the NYHA classification and PVR severity grade has not been investigated yet. One recent study showed that patients with more severe PVR showed less improvement in NYHA class at 6 months compared with patients with none or mild PVR.^4^ In our study, although patients with moderate PVR did not have a worse NYHA functional class at six months, this was observed at one year of follow-up. Nevertheless, such difference was not detected when only classes III and IV were considered, and this phenomenon needs to be further elucidated.

### Limitations

This was a retrospective, observational study with its obvious limitations. Our sample size was small, which limits the conclusions that can be drawn regarding clinical outcomes and analysis of predictors, with insufficient power to make firm conclusions especially about mortality. Besides, the existence of only one method available to evaluate PVR (echocardiography), quantification of PVR grade, technical difficulties, and the use of different assessment methods for different prosthesis should be considered. Echocardiographic analyses were not performed by an independent Core-lab, and not all patients had available echocardiographic data at the different times of follow-up. For this reason, a paired analysis was performed. The study proposes a hypothesis and suggests future research on the theme.

### Conclusion

PVR after TAVI remains a frequent condition, with differences in baseline clinical and echocardiographic characteristics between the groups of different severity. In our sample, the presence of moderate or severe PVR was not a predictor of mortality or rehospitalization due to cardiac causes in the medium term, which may be attributed to the improvement in regurgitation severity grade over the first year after TAVI. For future research, the authors believe that it is crucial to identify patients at higher risk of worsening or lack of improvement of PVR and its related mechanism, and to conduct a longer follow-up of these patients.
